# Home-based video-polysomnography for sleep-related motor behaviors: development, feasibility, and diagnostic performance at the Bologna Sleep Center

**DOI:** 10.1093/sleep/zsaf264

**Published:** 2025-09-04

**Authors:** Greta Mainieri, Luca Baldelli, Francesco Mignani, Francesca Cotichelli, Giuseppe Loddo, Filomena Miele, Ezechiele Foschini, Angelica Montini, Felice Di Laudo, Caterina Pazzaglia, Monica Sala, Federica Provini

**Affiliations:** IRCCS Istituto delle Scienze Neurologiche di Bologna, Bologna, Italia; IRCCS Istituto delle Scienze Neurologiche di Bologna, Bologna, Italia; Department of Biomedical and NeuroMotor Sciences, University of Bologna, Bologna, Italy; IRCCS Istituto delle Scienze Neurologiche di Bologna, Bologna, Italia; Department of Biomedical and NeuroMotor Sciences, University of Bologna, Bologna, Italy; Department of Primary Care, Azienda AUSL di Bologna, Bologna, Italy; IRCCS Istituto delle Scienze Neurologiche di Bologna, Bologna, Italia; Department of Biomedical and NeuroMotor Sciences, University of Bologna, Bologna, Italy; IRCCS Istituto delle Scienze Neurologiche di Bologna, Bologna, Italia; Department of Biomedical and NeuroMotor Sciences, University of Bologna, Bologna, Italy; Department of Biomedical and NeuroMotor Sciences, University of Bologna, Bologna, Italy; Department of Biomedical and NeuroMotor Sciences, University of Bologna, Bologna, Italy; IRCCS Istituto delle Scienze Neurologiche di Bologna, Bologna, Italia; IRCCS Istituto delle Scienze Neurologiche di Bologna, Bologna, Italia; IRCCS Istituto delle Scienze Neurologiche di Bologna, Bologna, Italia; Department of Biomedical and NeuroMotor Sciences, University of Bologna, Bologna, Italy

**Keywords:** home video-polysomnography, ambulatory video-polysomnography, sleep-related motor behaviors, sleep disorders, disorders of arousal, REM sleep behavior disorder

## Abstract

**Study Objectives:**

For most sleep disorders, in-laboratory video-polysomnography (VPSG) is currently considered the gold diagnostic standard. However, a growing need for more accessible diagnostic tools has been highlighted. This study aims to describe the experience of the Bologna Sleep Center in evaluating sleep-related motor behaviors using home VPSG.

**Methods:**

Consecutive patients referred to the Bologna Sleep Center between April 2016 and May 2024 for suspected sleep-related motor behaviors were recorded. Based on clinical suspicion, patients underwent either a 48-h monitoring with a full electroencephalogram montage (for non-rapid eye movement parasomnias or epilepsy) or a 24-h monitoring with a sleep montage (for patients with rapid eye movement [REM] sleep behavior disorder). Patients were equipped in the sleep lab by expert sleep technicians, who also provided instructions for continuing the recording in the home setting. A technical evaluation of recording quality was conducted on the first 50 recordings.

**Results:**

We included 305 patients, resulting in a total of 489 home VPSGs. Overall, 82% of the recordings were diagnostic (either confirming or excluding the clinical suspicion), while 18% were nondiagnostic due to insufficient evidence to confirm a diagnosis or technical issues. A detailed technical evaluation of the quality of the tracings in the first 50 recordings revealed a mean artifact percentage of 8% on polygraphic channels.

**Conclusions:**

Home VPSG demonstrated good diagnostic accuracy and exhibited limited technical issues that do not significantly interfere with its diagnostic capability. Recording in the patient’s natural environment may increase the likelihood of capturing habitual episodes.

**Statement of Significance**

The present study demonstrates that diagnosing sleep-related motor behaviors using home video-polysomnography is feasible. In particular, one recording night is often sufficient for a diagnosis of REM sleep behavior disorder, while two consecutive nights provide good diagnostic efficacy for disorders of arousal, for which the home environment may increase the likelihood of capturing habitual episodes. Careful work by the sleep lab staff is essential for the success of the recordings, ensuring tracings with minimal artifacts. Future studies may further minimize technical issues by incorporating video tutorials or telemonitoring to address critical technical challenges. In conclusion, home video-polysomnography may serve as a valuable implement at the sleep expert’s disposal, providing lower costs and shorter waiting lists than in-lab video-polysomnography.

## Graphical Abstract

**Graphical Abstract:**
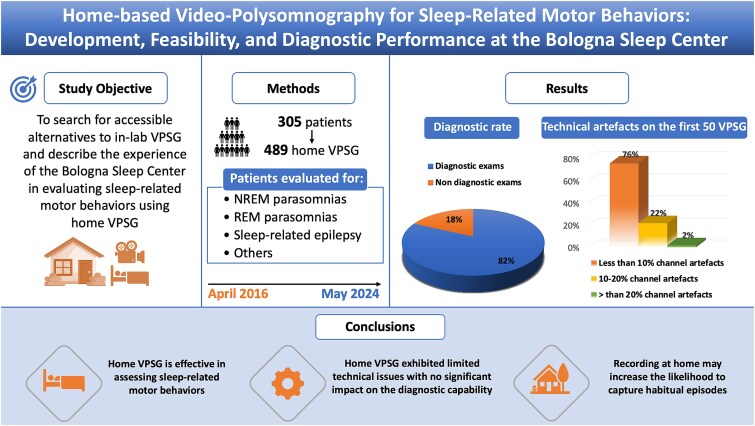

## Introduction

In sleep centers, various sleep disorders, ranging from insomnia to breathing disorders to parasomnias, are routinely evaluated and may require both subjective and objective measures for a definite diagnosis. Attended in-laboratory video-polysomnography (VPSG), classified as type I PSG, is the most accurate objective sleep assessment for most sleep disorders and is mandatory for the definitive diagnosis of some of them, according to the current International Classification of Sleep Disorders (3rd Ed-ICSD) [1, 2]. However, several studies have highlighted the practical limitations of in-lab PSG, including the need for the on-site presence of specialized sleep technologists, higher costs, and long waiting lists [3, 4]. In addition, the hospital setting may affect sleep quality, reduce the likelihood of capturing habitual episodes, and be usually impractical for children or the elderly [3].

For these reasons, other types of recordings, including type 2 unattended PSG, type 3 unattended polygraphy, and type 4 unattended recording, have become widely used, particularly for the diagnosis of sleep-related breathing disorders [5–7]. Type 2 unattended PSG is often conducted without video recording and still requires the sleep lab for the initial setup (placing the electroencephalogram [EEG] electrodes and initializing the recording), which takes approximately 1 h [3]. Unattended PSG has been used in large cohorts of the general population to study sleep structure and detect the prevalence of different sleep disorders [8–10]. However, in the study of nocturnal motor behaviors, mainly rapid eye movement (REM) and non-rapid eye movement (NREM) parasomnias or sleep-related epilepsies, video analysis, along with EEG and polygraphic parameter evaluation, is still essential for performing an accurate differential diagnosis. In the field of epilepsy, video-EEG telemetry at home has become widely used and is often integrated into the diagnostic procedure in tertiary care services [11–16]. Moreover, video-EEG telemetry has proven to be a valuable tool in providing continuous diagnostic services to patients with epilepsy during the recent COVID-19 pandemic [14].

Regarding sleep parasomnias, few studies have evaluated the efficacy of home-video recordings (in the absence of EEG and neurophysiological parameters) in the diagnosis or therapy monitoring of NREM parasomnias [17, 18], showing that the habitual environment may help capture more complex episodes than laboratory VPSG. To our knowledge, however, no study has evaluated the performance of ambulatory VPSG in the diagnosis of sleep-related behavior disorders. In the present study, we aim to describe the experience of the Bologna Sleep Center in the development, feasibility, and accuracy of home-based VPSG in the diagnostic evaluation of sleep-related motor behaviors.

## Methods

### Study sample

All patients included in the study were consecutively referred to the Sleep Center of the Department of Biomedical and NeuroMotor Sciences, University of Bologna, and IRCCS Istituto delle Scienze Neurologiche di Bologna, between April 2016 and May 2024, and performed a home VPSG. Patients were evaluated by sleep disorder specialists (F.P., G.L., G.M., L.B., and A.M.) and were assessed for sleep-related disorders, with the most common diagnostic suspicions being NREM and REM parasomnias, followed by a minority of suspicions of sleep-related epilepsy, rhythmic movement disorder, and psychogenic nocturnal episodes. All patients were offered a diagnostic home VPSG to confirm or exclude the diagnostic suspicion. Patients and/or caregivers who lacked cooperation, or patients unable to handle the recording equipment or who lacked electrical outlets at home were deemed ineligible for home VPSG and were not included in the study. All patients were instructed to maintain their usual sleep routine in the week preceding the evaluation. No sleep deprivation protocols were applied. Patients taking clonazepam or melatonin were advised to undergo slow and safe tapering at least 15 days before the recording, in order to increase the likelihood of capturing motor episodes. For patients with REM sleep behavior disorder (RBD) suspicion, whenever a possible temporal correlation with antidepressant use was identified, we tried to taper the drug before the recording. If this was not feasible, the medication was taken into account when defining the etiology of RBD. Based on the diagnostic suspicion, the clinician would choose between two different types of EEG montages, in addition to varying recording durations. Specifically, patients with suspected NREM parasomnia or epilepsy were scheduled for 48-h recordings with a complete 10-20 EEG montage. Conversely, suspicion of RBD was generally scheduled for 24-h recordings with a standard sleep montage with forearm EMG. The study was approved by the local ethical committee (no. 17036, no. 17176, and no. 21075), and each participant provided written consent.

### The procedure and equipment

On the scheduled date, patients were instructed to arrive at the sleep lab around 2:00 pm. They were equipped in the sleep lab by specialized sleep technicians (F.M., C.P., and M.S.). The recording equipment consisted of the XLTEK Trex HD (Natus Medical Incorporated) with a Handycam HDR-CX700 video camera (Sony, 12.3-megapixel resolution).

Video-polysomnographic recordings were performed using a standard bipolar EEG montage (according to the international 10-20 system) and included 19 electrodes (Fp1, Fp2, F3, F4, F7, F8, Fz, C3, C4, Cz, T3, T4, T5, T6, P3, P4, Pz, O1, and O2), as well as an electrocardiogram, electro-oculogram, chin and bilateral anterior tibialis electromyography, thoracoabdominal plethysmography bands, and synchronized audio–video recording for a complete montage. The sleep montage for suspected RBD included EEG channels (F3–M2, C3–M2, and O1–M2), electrocardiogram, electro-oculogram, chin, bilateral anterior tibialis, and wrist flexor/extensor electromyography, thoracoabdominal plethysmography bands, and synchronized audio–video recording.

After the setup, the technician performed biocalibration and checked for the absence of artifacts before initializing the recording. A detailed summary of montage features is shown in Box 1.

**Box 1:** Technical procedure.

Montage	Advantages and limits
The sleep technician applies EEG electrodes (1.5 m in length for scalp and 2.5 m in length for electrodes placed on the body) using collodion. To ensure a good-quality signal, for EEG electrodes the value of impedance is below 10 KΩ while for ECG and EMG is below 30 KΩ. All electrodes are positioned under the clothing and are then attached to the portable device placed in a bag that the patient wears over their shoulder.	*This electrode length is particularly useful for home video recordings to allow flexibility of movement.* *Collodion montage provides a good holding of electrodes for long-term monitoring* *The patient can easily undress to wear their pyjamas and freely move around the house* *Technical artifacts cannot be corrected and may invalidate the exam*
Video	Advantages and limits
When the recording is initialized, the video camera is synchronized with the recording trace. All instructions on how to handle the video camera are given to patients and/or caregivers and a simulation is performed. A tripod to support the camera and ensure a good framing of the patient in the bed is given to patients and/or caregivers. To switch on the video camera, the patient must press three buttons: start, nocturnal modality, and infrared light. The video camera has to be connected to power supply.	*Video recording is restricted to time periods of interest (nocturnal sleep or diurnal naps)* *The memory card of the camera allows the recording of two nights* *The nocturnal modality and infrared light allow to record with high definition in the dark* *Technical problems with video camera or patient’s incorrect management (wrong framing, lack of nocturnal modality, etc.) may invalidate the exam*
Battery replacement	Advantages and limits
The patient must replace the batteries after the first 24 h of recording, both on the recording device and on the infrared spotlight.	*The recorder has no buttons, and no procedure is required to restart it after replacing the batteries. The recording continues with the initialized settings* *If the patient forgets to replace the batteries, the second night of recording is lost*

ECG, electrocardiogram; EEG, electroencephalogram; EMG, electromyography.

For patients undergoing 48-h recordings, patients and/or caregivers were instructed to replace the batteries after the first night to prevent recording interruptions beyond 24 h. Patients were also instructed on the proper functioning and positioning of the video camera when going to bed. To optimize visibility of nocturnal episodes, the video camera had to be placed at the foot of the bed at a higher elevation to ensure a full-body view and had to remain connected to the power supply. To further enhance night time visibility, patients were instructed to switch on the infrared lights and, if possible, sleep uncovered while wearing dark clothing (as white clothing increases video reflection). An informative document containing both written and illustrated instructions was provided to each patient and/or caregiver.

In addition, patients were given a sleep diary to document the occurrence of habitual episodes (with the aid of a sleep partner or caregiver, if present), the timing of events, and general details regarding sleep quality, lights-off time, and subjective sleep latency.

After completing the home VPSG, each patient returned to the sleep laboratory for electrode removal. The recording was then transferred and downloaded onto a designated computer equipped for analysis.

All recordings were reviewed by neurologists experts in sleep medicine (F.P., G.M., and G.L.). Sleep stages and events were scored according to American Academy of Sleep Medicine (AASM) criteria [19]. All nocturnal behavioral episodes recorded were carefully identified and analyzed to establish a diagnosis. In patients with RBD, motor events were classified as RBD episodes according to Cesari et al. [20], while disorder of arousal (DoA) episodes were classified based on Loddo et al. [21].

### Technical quality of the recordings

In order to verify the reliability of the system, the first 50 consecutive recordings underwent a quality inspection by expert sleep technicians (E.F., Fi.M.). Specifically, a quality analysis of the polygraphic tracings was conducted by inspecting and quantifying the number of artifacts, the absolute number of channels with artifacts, and the relative percentage of affected channels over the total number of channels. This analysis was performed on three consecutive 30-s epochs every 15 min of recording.

The inspected channels included all electroencephalographic channels, the electro-oculogram, electromyographic (EMG) channels (mylohyoid and anterior tibialis), electrocardiographic lead, mastoid, thoracoabdominal respiratory belt, and microphone.

### Statistical analysis

For each exam, there was an initial clinical suspicion, which could involve a single sleep condition, diagnostic uncertainty between two disorders, or the concurrent presence of both. The nocturnal home recording was considered diagnostic if:

a) it confirmed the initial suspicion; or
b) it excluded the initial suspicion by revealing another sleep disorder (a mimic of the clinical suspicion) or resulted in a normal finding—the latter being applicable only in cases of suspected RBD, where normal REM sleep atonia excludes the diagnosis.

In addition, the home VPSG was considered nonevaluable in cases where technical issues arose (e.g. EEG/EMG artifacts that prevented proper interpretation, missing video recording, interrupted recording) or when patients had insufficient REM sleep (defined as less than 15 min in duration [20]). Specifically:

- For NREM parasomnias, the recordings were considered diagnostic if at least one usual episode was recorded. If no episodes were recorded, the exam was considered nondiagnostic.
- For sleep-related epilepsy, the exam was deemed diagnostic if at least a seizure was recorded or also if clear epileptic discharges or interictal epileptic abnormalities were present.
- For RBD suspicion, exams could be either diagnostic or nonevaluable. The exam was considered diagnostic if a habitual episode or the presence of REM sleep without atonia (RSWA) was detected [1, 19] (confirming the suspicion) or if normal REM sleep atonia was observed (excluding the suspicion and suggesting a normal exam or a possible RBD mimic, such as sleep apnea or periodic limb movements).
- For other suspected conditions, including nightmares, psychogenic nonepileptic seizures (PNES), rhythmic movement disorder, the exam was considered diagnostic if at least one episode was recorded.

Statistical analysis was performed using STATA 18 software packages (version 18.0). Qualitative variables were expressed as percentages while quantitative variables as mean ± standard deviation (SD). Pearson chi-squared test (χ^2^) or Fisher’s exact test were employed to examine categorical variables; comparison between means were performed using unpaired or paired *t* test for parametric data and Wilcoxon rank-sum (Mann–Whitney) test for nonparametric data. A *p* < .05 was set as level of significance. Cohen’s *D* and Cramér’s *V* were calculated as measures of effect size for quantitative and categorical variables, respectively.

## Results

### Diagnostic efficacy and technical issues of home video-polysomnography

**Figure 1:**
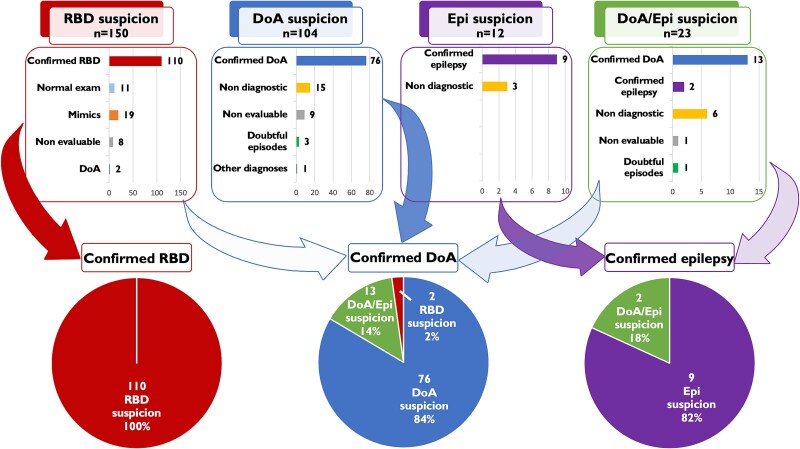
Entrance and final diagnoses. The upper part of the figure presents the initial diagnostic suspicions for various sleep conditions. The bar chart illustrates the final diagnoses for each suspicion, including the number of nondiagnostic and nonevaluable exams. In particular, in RBD suspicion the exam was diagnostic in 95% of cases, including a confirmed diagnosis of RBD or the exclusion (normal exam or presence of other sleep disorders mimicking a RBD). In the lower part of the figure, pie charts depict the final number of patients diagnosed with RBD, DoA, and epilepsy, along with their respective origins from the initial diagnostic suspicions. The intensity of the arrow colors corresponds to the proportion of patients with a specific final diagnosis, originating from the various diagnostic suspicions’ subgroups. RBD, REM sleep behavior disorder; DoA, disorders of arousal; epi, epilepsy.

A total of 305 patients (189 males, mean age 50.1 ± 21.1 years) were included in this study, with a total of 489 recording days. Overall, the exam was diagnostic in 82.3% of patients (Table 1). The principal final diagnoses were DoA and RBD. The initial suspicions and final diagnoses are illustrated in Figure 1. In addition, among the six suspected cases of SRED, five were confirmed while one remained nondiagnostic; of the four suspected cases of sexsomnia, two were deemed nondiagnostic and two nonevaluable; out of three PNES suspicions, two were confirmed and one was nondiagnostic; the single suspected case of body rolling was confirmed, whereas both the suspected nightmare and nocturnal panic attack cases resulted in nondiagnostic outcomes. Overall, 14.4% of patients were taking antidepressants, 8.2% were on levodopa or dopamine agonists, a minority (5.2%) used antiepileptic drugs, and 2.0% were taking antipsychotics.

**Table 1:** Diagnostic efficacy and technical issues: difference between NREM parasomnia/epilepsy and RBD suspicions

	Total VPSGs	NREM parasomnia/epilepsy suspicion	RBD suspicion	*P* value	Cramér’s *V*/Cohen’s *D*
Patient number	305	155	150		
Sex ratio (male)	189 (61.97%)	75 (48.39%)	114 (76.00%)	**<.0001**	−0.28^a^
Mean age (mean ± SD)	50.1 ± 21.1	33.43 ± 15.46	67.44 ± 8.53	**<.0001**	−2.71^b^
Recorded nights					
*24 h*	121 (39.67%)	17 (10.97%)	104 (69.33%)		
*48 h*	184 (60.33%)	138 (89.03%)	46 (30.67%)		
Diagnostic efficacy					
* Diagnostic recordings (n, %)*	251 (82.30%)	109 (70.32%)	142 (94.67%)	**<.0001**	0.32^a^
* Not diagnostic recordings (n, %)*	54 (17.70%)	46 (29.68%)	8 (5.33%)	**<.0001**	
Technical issues	51 (16.39%)	33 (21.29%)	18 (12.00%)	**.030**	−0.12^a^
* Patient related*	35 (68.63%)	24 (72.73%)	11 (61.11%)	.393	−0.09^a^
* Equipment related*	16 (31.37%)	9 (27.27%)	7 (38.89%)		

Quantitative variables are expressed as mean ± standard deviation. Qualitative variables are expressed as absolute number and percentage. NREM, non-rapid eye movement; RBD, REM sleep behavior disorder; VPSG, video-polysomnography. Bold represents significant difference (*p* <.05). Italic represents subcategories. ^a^Cramér’s *V*; ^b^Cohen’s *D*.

When considering the two main diagnostic suspicion groups (NREM parasomnia/epilepsy suspicion vs RBD), the exam was significantly more diagnostic in the RBD suspicion subgroup (94.67% of recordings, *p* < .0001). The small proportion of nondiagnostic exams in the RBD suspicion group (5.33%) was due to unevaluable recordings, either because of insufficient REM sleep time or technical artifacts in the polysomnography channels that prevented RSWA scoring (Figure 1).

In the NREM parasomnia/epilepsy suspicion subgroup, diagnostic confirmation required the documentation of a habitual episode. As a result, a higher proportion of nondiagnostic exams was observed (29.68% of recordings, *p* < .0001), primarily due to the absence of recorded episodes. The specific diagnostic outcomes of the two main subgroups are illustrated in Figure 1.

The overall occurrence of technical problems was slightly higher in the NREM parasomnia/epilepsy suspicion group (*p* = .030), but this difference was not significant when considering only patient-related issues. In addition, technical problems invalidated the exam, limiting the diagnostic rate, in only 5.2% of cases (16/305). The different types of technical problems are listed in Supplementary Table S1.

### Principal diagnoses

The two main final diagnoses were DoA and RBD. A total of 29.8% of patients (91 out of 305) were diagnosed with DoA. In 11 patients, the second night of recording was unavailable, primarily due to the premature interruption of the recording (mostly caused by failure to switch to new batteries). However, the first recording night was still sufficient to capture at least one habitual episode and confirm the diagnosis.

In Table 2, we compared the sleep and episode data between the first and second nights in DoA patients who completed the full 48-h assessment (*n* = 80). Patients with DoA tended to sleep longer on the first night of recording (*p* < .0001), with a higher number of sleep cycles (*p* = .0001) and a consequent increase in the absolute duration of all sleep stages (*p* < .0001 for N1, N2, and REM sleep minutes), except for the N3 stage, whose absolute duration remained stable over both nights. Conversely, the proportion of N3 sleep was significantly higher during the second night of recording (*p* = .0002), likely due to the shorter total sleep time. Despite a higher wake after sleep onset during the first recording night (*p* = .0002), sleep efficiency did not differ between the two nights. Finally, a slightly higher number of episodes was recorded during the second night (*p* = .036).

**Table 2:** Differences in sleep and episode features in DoA subjects between the first and second recording nights

	VPSG DoA First night	VPSG DoA Second night	*P* value	Cohen’s *D*
VPSG with 48 h sleep available	80	80		
Sleep data				
* TST (min)*	474.7 ± 95.6	406.5 ± 85.0	**<.0001**	0.68
* SL (min)*	8.9 ± 9.9	10.7 ± 9.7	.056	−0.21
* SE (%)*	89.2 ± 7.5	89.2 ± 6.9	.912	0.01
* REM sleep latency (min)*	88.4 ± 40.5	87.1 ± 39.0	.823	0.02
* WASO (min)*	40.3 ± 26.9	29.5 ± 18.3	**.0002**	0.45
* N1 (%)*	7.5 ± 3.7	6.9 ± 3.8	.080	0.20
* N2 (%)*	43.5 ± 8.7	43.1 ± 7.5	.649	0.04
* N3 (%)*	25.0 ± 8.7	28.1 ± 7.9	**.0002**	−0.42
* REM (%)*	23.8 ± 4.8	21.8 ± 5.4	**.0010**	0.38
* N1 stage (min)*	35.1 ± 17.2	28.6 ± 17.1	**<.0001**	0.49
* N2 stage (min)*	207.1 ± 60.8	174.5 ± 45.9	**<.0001**	0.57
* N3 stage (min)*	117.7 ± 41.7	113.8 ± 41.3	.253	0.13
* REM stage (min)*	113.8 ± 36.2	89.9 ± 31.3	**<.0001**	0.63
* Sleep cycles (mean ± SD)*	4.7 ± 1.3	3.9 ± 1.1	**.0001**	0.47
* Episodes per night (mean ± SD)*	2.60 ± 2.36	3.20 ± 2.71	**.036**	−0.25

Results are expressed as mean ± standard deviation. DoA, disorders of arousal; min, minutes; NREM, non-rapid eye movement; REM, rapid eye movement; SE, sleep efficiency; SL, sleep latency; TST, total sleep time; VPSG, video-polysomnography; WASO, wake after sleep onset. Bold represents significant difference (*p* <.05). Italic represents subcategories.

A total of 3.6% of patients (11 out of 305) received a confirmed diagnosis of epilepsy. The majority of these cases (81.8%) originated from the subgroup with a specific suspicion of epilepsy, while the remaining two were identified within the subgroup with mixed DoA/epilepsy suspicion (Figure 1). A total of 36.1% of patients (110 out of 305) were diagnosed with RBD. Overall, a complex episode was recorded in 44.5% of patients, while in the remaining cases RSWA was observed. In addition, in 8.1% of patients, video was unavailable, making it impossible to determine whether a complex episode had been recorded. However, a diagnosis of RBD was possible in these cases based on clinical history and valid polygraphic quantification of RSWA, despite the lack of video. All patients diagnosed with RBD based on RSWA met the quantitative threshold of more than 32% [22] and, in particular, exhibited a mean RSWA of 61.74% ± 19.63%.

In Table 3, we compared patients with and without the recording of a complex episode (excluding patients with unavailable video, as it was impossible to determine if a complex episode was captured). No significant differences were found between the two groups, except for a significantly higher presence of isolated RBD (*p* = .048) and a trend toward a higher proportion of REM sleep (*p* = .056) in patients with a recorded complex RBD episode.

**Table 3:** Sleep and clinical features distinguishing patients with RBD who do or do not have recorded complex episodes

	Patients with RBD episode	Patients with RSWA	*P* value	Cramér’s *V*/ Cohen’s *D*
Number	49	52		
Sex (male)	40 (81.63%)	38 (73.08%)	.305	0.10^a^
Mean age (mean ± SD)	68.18 ± 7.05	67.71 ± 8.08	.756	0.06^b^
Etiology				
* Isolated*	34/47 (72.34%)	27/51 (52.94%)	**.048**	0.20^a^
* Secondary*	13/47 (27.66%)	24/51 (47.06%)		
Sleep data				
* TST (min)*	376.02 ± 95.50	390.47 ± 113.34	.491	−0.14^b^
* SL (min)*	23.48 ± 55.41	16.10 ± 15.60	.358	0.18^b^
* SE (%)*	75.80 ± 13.30	76.25 ± 12.29	.863	0.04^b^
* REM sleep latency (min)*	118.57 ± 62.90	117.58 ± 83.69	.946	0.01^b^
* WASO (min)*	72.21 ± 39.40	69.44 ± 36.40	.717	0.07^b^
* N1 (%)*	13.61 ± 8.43	16.11 ± 9.35	.164	−0.28^b^
* N2 (%)*	39.63 ± 10.17	38.86 ± 11.51	.725	0.07^b^
* N3 (%)*	26.27 ± 10.65	27.62 ± 12.27	.560	0.12^b^
* REM (%)*	20.51 ± 8.17	17.40 ± 7.96	.056	0.39^b^
* N1 stage (min)*	49.97 ± 24.96	57.30 ± 27.23	.165	−0.28^b^
* N2 stage (min)*	161.46 ± 71.17	152.16 ± 64.37	.494	0.14^b^
* N3 stage (min)*	99.19 ± 44.04	112.74 ± 73.96	.273	−0.22^b^
* REM stage (min)*	79.57 ± 37.01	68.20 ± 35.66	.121	0.31^b^
* Sleep cycles (mean ± SD)*	3.27 ± 1.16	3.38 ± 1.26	.657	−0.09^b^
* PLM index (mean ± SD)*	38.22 ± 29.80	37.31 ± 32.64	.906	0.03^b^

Results are expressed as mean ± standard deviation. Qualitative variables are expressed as absolute number and percentage. RBD, REM sleep behavior disorder; RSWA, REM sleep without atonia; TST, total sleep time; min, minutes; SL, sleep latency; SE, sleep efficiency; WASO, wake after sleep onset; PLM, periodic limb movement. Bold represents significant difference (*p* <.05). Italic represents subcategories. ^a^Cramér’s V; ^b^Cohen’s D.

### Quality evaluation of the first 50 recordings

Supplementary Figure S1 illustrates the evaluation of the percentage of channel artifacts in the first 50 consecutive recordings as well as the trend of mean artifact percentage over time. Overall, 76% (38 out of 50) of PSG recordings had less than 10% channel artifacts, while 22% (11 out of 50) had a mean artifact percentage between 10% and 20%. Only one recording had an artifact percentage exceeding 20%.

At the end of the first 24 h, the mean artifact percentage ranged between 9% and 10%, corresponding to approximately two channels affected by artifacts. This percentage increased over time, reaching a maximum of 16% at the end of 48 h of recording.

The most affected channels were as follows: EOG with 25% artifacts, ECG with 24%, and mylohyoid EMG with 13%. EEG channels were less impacted, with 9% artifacts. Additional artifact percentages were observed in the mastoid (10%), thoracoabdominal respiratory belts (10%), anterior tibialis EMG (7%), and microphone (2%).

## Discussion

To our knowledge, this is the first description of the diagnostic efficacy and feasibility of home VPSG in a large cohort of patients with sleep-related motor behaviors. Our study demonstrates that home VPSG proved to be a reliable diagnostic procedure, well-tolerated by patients, with minimal technical issues. When dealing with paroxysmal events, a critical challenge is ensuring that the equipment can effectively capture the neurophysiological and behavioral features of an event in an unsupervised environment [16]. In contrast to epilepsy studies, this issue is less pronounced in our study since the events of interest are primarily restricted to nocturnal sleep, which presents several advantages. First, patients remain still for most of the night, ensuring stable polygraphic recordings with minimal artifacts, which typically do not impact the final outcome (Supplementary Figure S1). In addition, when the camera is securely fixed and correctly positioned by the patient, it provides optimal visibility for sleep-related events. In our study, technical issues—whether related to the equipment or the patient—occurred in a small number of exams, supporting the good diagnostic efficacy of home VPSG in evaluating sleep-related motor behaviors. In light of the promising outcomes observed in the field of epilepsy [16], home-based VPSG may therefore hold significant potential for broader application in the assessment and management of sleep-related motor behaviors.

Our findings suggest that the habitual home environment may increase the likelihood of capturing a habitual episode, even under basic sleep–wake conditions. In fact, prolonged home-video recordings have shown that more complex and frequent DoA episodes are captured in the home setting [17, 18]. In our study, home VPSG demonstrated a 70% diagnostic efficacy in NREM parasomnia or epilepsy suspicion group. When considering only DoA suspicion (Figure 1), the confirmation rate was similar, with 73% (76 out of 104) of cases confirmed. This result falls within the range of diagnostic efficacy (30%–100%) [23–36] previously reported in laboratory-based studies. Excluding studies with sleep deprivation protocols, which increase the likelihood of capturing a usual episode [27], and considering adult studies similar to ours (but performed in the sleep laboratory) [24–26, 32], the diagnostic efficacy of our sample was comparable to some series (76.5% [26] and 67.3% [32]) and higher than others (59% [25] and 31.4% [24]). Although VPSG is not currently required for the diagnosis of DoA, it is frequently performed in adult populations, primarily for differential diagnostic purposes and to rule out comorbid sleep disorders. In our DoA sample, patients tended to fall asleep more quickly and slept longer during the first night of recording, when they could sleep ad libitum and were not subject to their school or work schedules. This finding may have been influenced by the necessity to travel back to the sleep lab after the second night of recording, which resulted in a forced awakening. However, the total amount of N3 sleep was comparable between the first and second nights, suggesting a preserved and stable slow-wave sleep homeostasis in DoA. In addition, even if sleep efficiency was equivalent between the first and second nights, indicating no “first night effect,” the number of recorded DoA episodes was significantly higher during the second recording night (Table 2). This suggests that the second night may be even more representative of a habitual night than the first. Despite these speculations, a second night of recording seemed to enhance the diagnostic efficacy in DoA. To this extent, we cannot exclude that the higher likelihood of capturing a DoA episode may be related to the prolonged observation time, regardless of being at home or in the lab. Since the primary aim of our study was to evaluate the diagnostic performance of home VPSG—particularly its ability to capture video-documented episodes and produce clean, analyzable polygraphic data, we employed a conservative diagnostic criterion, classifying patients as DoA-positive only if at least one episode was captured. However, incorporating EEG indices such as spontaneous arousals from N3 [25] may further improve diagnostic yield and allow stratification into definite and probable DoA diagnoses [23].

The diagnostic efficacy in the RBD suspicion group was approximately 95%. Specifically, the suspicion was confirmed in 77% of patients, while in a minority the diagnosis was excluded (either due to a normal exam or the presence of RBD mimics). This result is difficult to compare with existing literature, as no home VPSG studies are available, and most sleep center studies on RBD have focused on patients with an already confirmed diagnosis. These studies primarily assessed the diagnostic sensitivity of VPSG using different methodologies, such as motor activity detection on the chin, the Sleep Innsbruck Barcelona (SINBAR) method, automatic muscle activity detection, and video analysis [22, 37–41]. However, our RBD confirmation rate is comparable to some studies in which sleep experts confirmed RBD in 75% [42] and 66% [43] of suspected cases, respectively. Overall, most studies agree that a single night is sufficient for RBD diagnosis [40, 44], that video inspection combined with motor activity analysis enhances diagnostic efficacy [40], and that a second night provides only moderate additional information [45]. The findings of our study reinforce this notion, demonstrating that a single night of home VPSG in suspected RBD cases is sufficient to establish a diagnosis in approximately 95% of cases, either confirming or ruling out the condition.

Regarding complexity of RBD episodes, while some efforts have been made to classify motor events in RBD [44, 46], no expert consensus currently exists, although proposals have been advanced to standardize RBD diagnosis and potential phenotypes [20]. In approximately half of our patients, we were able to record a complex RBD episode with clear dream enactment. This is similar to findings from a previous laboratory study [47] but represents a higher proportion than other studies, where major motor manifestations were observed in only one-fifth of the study sample [40]. Several factors may account for these differences. First, while tonic motor activity remains stable between nights, phasic activity and motor behaviors may exhibit inter-night variability [44]. Indeed, night-to-night variability in phasic motor activity may have influenced our findings, particularly in individuals with suspected RBD but without significant RSWA on the recorded night, potentially leading to an underestimation of RBD prevalence. Second, the amount of REM sleep recorded plays a crucial role. In laboratory studies, the absence of REM sleep has been attributed to a possible “first-night effect” [48]. In contrast, in our home-based VPSG study, recordings with insufficient REM sleep were negligible compared to lab series [48, 49]. In addition, patients who exhibited complex RBD episodes had a higher proportion of REM sleep (Table 3), which is expected, as increased REM sleep duration likely raises the probability of capturing complex episodes. A third consideration is the etiology of RBD. Our sample included both isolated and secondary RBD cases, and we found that patients with complex RBD episodes were significantly more likely to have an “isolated” etiology. This supports previous findings suggesting that the frequency and intensity of complex motor manifestations in RBD tend to diminish over time in patients with secondary RBD [50]. Finally, home VPSG showed a very good diagnostic efficacy with few technical issues in this group, despite the fact that it included elderly patients and individuals with neurodegenerative diseases—both factors that could have potentially undermined the feasibility of home VPSG. We believe this was avoided mainly due to the single-night recording design, which minimized technical complications such as battery replacement, and the constant presence of caregivers, which allowed the delivery of clear instructions both verbally and through an illustrated leaflet.

The main limitation of our study is the lack of a laboratory control group with matched diagnostic suspicions or a protocol that included the same patients recorded both in the lab and at home. Such a design would have allowed for a direct comparison of diagnostic efficacy, as well as a comparison of sleep quality between the lab and the habitual home environment. However, considering the routine difficulties associated with standard clinical practice—exponentially increased by the recent COVID-19 pandemic—this approach would have required considerable effort from both patients and sleep lab staff, including technicians and clinicians. Future studies could consider matched-cohort designs or, preferably, crossover designs to assess both sleep structure and motor behaviors in laboratory versus home settings. In addition, we acknowledge that part of the favorable technical outcomes in our study may be attributed to the use of collodion-based EEG montages, which are typically available only in specialized settings. This limits the generalizability of our findings to broader clinical contexts. Furthermore, while particular attention was paid to tapering medications most directly involved in the control of DoA and RBD episodes (clonazepam for both and melatonin for RBD), and no patients were taking these drugs at the time of recording, we cannot completely exclude the influence of other psychotropic medications on sleep structure of our sample.

## Conclusions and Further Directions

In conclusion, home VPSG may serve as a valuable additional tool at the sleep expert’s disposal, providing several advantages such as significantly lower costs, reduced burden on healthcare facilities, and shorter waiting lists for patients in need of diagnostic evaluation. Home VPSG also increases accessibility for individuals who may face challenges traveling to a sleep lab, consequently broadening the availability of sleep-related diagnostic services. Moreover, this type of exam allows flexible scheduling based on clinical priorities, giving precedence to patients with violent, traumatic, or very frequent episodes, as well as accommodating those requiring drug tapering. Home VPSG demonstrated good diagnostic efficacy and limited technical difficulties in assessing sleep-related motor behaviors. To further reduce the probability of technical issues related to patients, future studies should consider the possibility of telemedicine communication with the sleep staff especially at key stages (e.g. before going to sleep to ensure correct video-camera installation, and during the second day of recording to remind patients to replace batteries). In addition to the illustrated brochure, video tutorials outlining all the steps that patients need to follow could be provided by the sleep staff to help avoid nondiagnostic exams. The diagnostic procedure was well-tolerated by patients, who were able to move freely in their habitual environment. In addition, by allowing patients to undergo monitoring in the comfort of their own homes, home VPSG can help preserve their normal sleep–wake routines, without being subject to the technician’s work schedule, which may improve the detection of specific sleep features as well as increase the likelihood of capturing habitual nocturnal episodes.

## Supplementary Material

Supplementary_Table_S1-Figure_S1_zsaf264

## Data Availability

The data underlying this article cannot be shared publicly due to the privacy of individuals who participated in the study. The data will be shared upon reasonable request to the corresponding author.
