# Supplementary material for: Home-based video-polysomnography for sleep-related motor behaviors: development, feasibility, and diagnostic performance at the Bologna Sleep Center
Source: Sleep. 2025 Sep 4;49(4):zsaf264. doi: 10.1093/sleep/zsaf264 (PMC13089511; doi:10.1093/sleep/zsaf264)
Supplement: Supplementary_Table_S1-Figure_S1_zsaf264 [file supplementary_table_s1-figure_s1_zsaf264.docx]

**Supplemental Materials**

**Home-based Video-Polysomnography for Sleep-Related Motor Behaviors: Development, Feasibility, and Diagnostic Performance at the Bologna Sleep Center**

**Greta Mainieri^1^, Luca Baldelli^1,2^*****, Francesco Mignani^1^, Francesca Cotichelli^2^, Giuseppe Loddo^3^, Filomena Miele^1,2^, Ezechiele Foschini^1,2^, Angelica Montini^2^, Felice Di Laudo^2^, Caterina Pazzaglia^1^, Monica Sala^1^, Federica Provini^1,2^**

^1^ IRCCS Istituto delle Scienze Neurologiche di Bologna, Bologna, Italia

^2^ Department of Biomedical and NeuroMotor Sciences, University of Bologna, Bologna, Italy

^3^ Department of Primary Care, Azienda AUSL di Bologna, Bologna, Italy

*Corresponding author

Luca Baldelli

IRCCS, Institute of Neurological Sciences of Bologna

Bellaria Hospital, Via Altura, 3 40139 Bologna (ITALY)

E-mail: luca.baldelli4@unibo.it

**Table S1. Technical problems in NREM parasomnia/epilepsy and RBD suspicion groups**

|  | **Overall technical problems (n=51)** | **NREM parasomnia/epilepsy suspicion** | **RBD suspicion** |
| --- | --- | --- | --- |
| **Exam related** | 16 | 9 | 7 |
| *Interruption of recording* | *5 (31.25%)* | *3 (33.33%)* | *2 (28.57%)* |
| *Desynchronization video-EEG* | *5 (31.25%)* | *4 (44.45%)* | *1 (14.29%)* |
| *Polygraphic artefacts* | *3 (18.75%)* | *0 (00.00%)* | *3 (42.85%)* |
| *Not downloadable recording* | *2 (12.50%)* | *1 (11.11%)* | *1 (14.29%)* |
| *Spotlight malfunctioning* | *1 (6.25%)* | *1 (11.11%)* | *0 (0.00%)* |
| **Patient related** | 35 | 24 | 11 |
| *Video not switched on (totality or a part of the recording)* | *13 (37.14%)* | *7 (29.17%)* | *6 (54.55%)* |
| *Unchanged batteries* | *10 (28.57%)* | *9 (37.50%)* | *1 (9.09%)* |
| *Infrared light not switched on* | *9 (25.72%)* | *6 (25.00%)* | *3 (27.27%)* |
| *Camera not connected to power supply* | *2 (5.71%)* | *2 (8.33%)* | *0 (00.00%)* |
| *Incorrect positioning of camera* | *1 (2.86%)* | *0 (0.00%)* | *1 (9.09%)* |

Qualitative variables are expressed as absolute number and percentage.


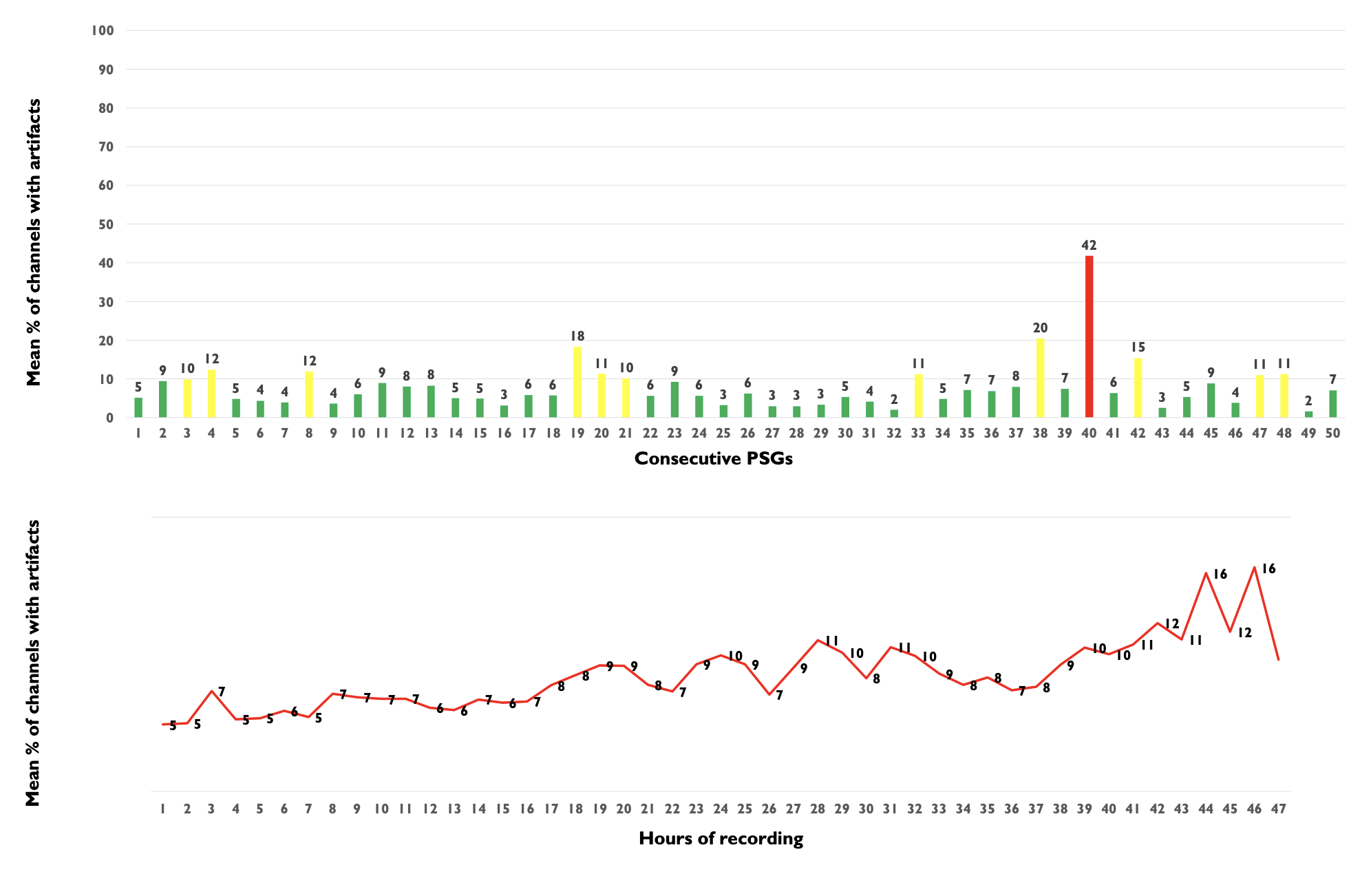


**Figure S1. Technical artefacts in the first 50 consecutive Home VPSGs**

The top panel displays the percentage of channel artefacts in the first 50 consecutive home VPSGs. Polygraphic recordings with less than 10% channel artefacts are shown in green, while those with a mean artefact percentage between 10% and 20% are shown in yellow. Exams with an artefact percentage exceeding 20% are indicated in red.

In the bottom panel, numbers above the red line represent the mean artefact percentage over time.
